# Factors in Randomized Controlled Trials Reported to Impact the Implementation of Patient-Reported Outcome Measures Into Routine Care: Protocol for a Systematic Review

**DOI:** 10.2196/14579

**Published:** 2019-11-26

**Authors:** Natasha Anne Roberts, Kimberly Alexander, David Wyld, Monika Janda

**Affiliations:** 1 School of Nursing Queensland University of Technology Kelvin Grove Australia; 2 Cancer Care Services Royal Brisbane and Women's Hospital Herston Australia; 3 School of Medicine University of Queensland St Lucia Australia; 4 Centre for Health Services Research University of Queensland Woolloongabba Australia; 5 Queensland University of Technology Kelvin Grove Australia

**Keywords:** patient reported outcomes, PROs, PROMs, clinical practice, implementation, implementation science, iPARIHS

## Abstract

**Background:**

Patient-reported outcome measures (PROMs) are tools that enable patients to directly report their own assessments of well-being, or symptoms, in a structured and consistent way. Despite the usefulness of PROMs in optimizing health outcomes, their use in clinical practice is not routine. PROMs are complex to integrate into the clinical setting, with many elements potentially impacting on the success of implementation. For this reason, a protocol has been developed to guide a systematic review to collate information on implementation as presented in the randomized controlled trials (RCTs) to date.

**Objective:**

The primary objective of this systematic review is to identify and synthesize factors available from RCT data about the fidelity of PROM interventions in clinical practice. The secondary objective will be an assessment of how implementation factors impact fidelity outcomes.

**Methods:**

Preferred Reporting Items for Systematic Reviews and Meta-Analyses reporting standards will be followed. MEDLINE, EMBASE, and the Cumulative Index to Nursing and Allied Health Literature via OvidSP will be accessed using a defined search strategy. Grey literature and ClinicalTrials.gov will be reviewed for unpublished studies. Data extraction will be done to identify fidelity and factors impacting implementation, summarized using a narrative synthesis. An evidence-based implementation science framework will assist in identifying potential elements of importance and their effect on the process and outcomes of implementation. A meta-analysis to assess the impact of implementation factors will be attempted. A Cochrane risk of bias tool will be used.

**Results:**

This protocol has received funding, and searches of databases will commence at the end of May 2019. It is planned that this systematic review will be finalized for publication in (December) 2019.

**Conclusions:**

Applying an implementation science evidence-based framework to the published literature may identify factors present in the data that impact on the implementation of PROMs into routine clinical care. This systematic review aims to improve understanding of how these factors impact the fidelity of this intervention, so that PROMs can be more effectively used in the care of patients. This systematic review can also offer more detailed information about the process and outcomes of successful implementation of PROMs.

**International Registered Report Identifier (IRRID):**

PRR1-10.2196/14579

## Introduction

### Background

Patient-reported outcome measures (PROMs) offer the potential [1] to provide unique information that can be used by health professionals [2] to optimize both the health care pathway and outcomes for patients [3]. PROMs are valid and reliable assessment tools that collect information directly from patients and are defined by the Food and Drug Administration as “any report of the status of patient’s health condition that comes directly from the patient, without interpretation of the patient’s response by a clinician” (p. 508) [4]. The benefits that come from using PROMs are informed by the response to this information actioned by the health professionals, not by the completion of the report alone. With ever-increasing health care costs, the use of PROMs may optimize patient care, service delivery, and patient outcomes. They may also identify problems that have previously gone unnoticed and lead to increased service use [5]. Hospitals are moving to integrate PROMs into clinical settings, both for individual patient care and quality improvement activities as well as allocation of resources [5]. However, PROMs need to be successfully implemented in a health care setting so that patients and clinicians can use them as intended.

An understanding of what is impacting implementation can ensure the optimal use of a PROM intervention. This remains problematic, however, and despite the large body of research on the benefits of using PROMs for patient care [5-7], the uptake of these tools remains slow, indicating that their implementation may be ineffective if not done well, with a reason for poor implementation grounded in the complexity of health care systems [8].

The difficulties of implementation have been acknowledged in the literature. Guidelines developed by leading PROMs academics and published by the International Society of Quality of Life (ISOQOL) offer a tailored evidence-based approach to implementing PROMs into clinical practice by identifying the needs of the institution. In 2016, Porter et al [4] reviewed and evaluated the factors impacting on PROM implementation to develop a framework that specified the elements that should be considered in PROM implementation. The challenges identified in the study by Porter et al [4] focused largely on the disconnect between PROMs data and clinical pathways. Other systematic reviews of qualitative and nonrandomized research reported that the setting into which the PROMs were to be integrated had a significant effect on their implementation [4,9]. Antunes et al (2014) [10] conducted a systematic review on the use of PROMs in palliative care and identified that slow uptake was likely because of staff and setting-related elements. They proposed a need for ongoing coordination and education during implementation [10]. These are similar to the findings of the systematic review by Duncan et al [11] of the facilitators and barriers of PROMs use by allied health professionals. They attributed the contextual factors, such as organizational and peer support, as having a significant impact on the success of PROM integration. Boyce et al [1] performed a systematic review of qualitative data describing staff experiences of PROM use and found that PROM usage was impacted if there was a lack of infrastructure to support staff in analyzing and acting on PROM data. What is consistently highlighted across these reviews is that the clinical setting and its stakeholders determine implementation success. A rapid scoping review of systematic reviews [12] was conducted to explore what evidence-based data were available regarding the implementation of PROMs. This scoping review demonstrated that there were some common factors impacting implementation of PROM, specifically, the engagement of staff, the use of technology, and pathways to respond to PROMs data.

None of the previous reviews, however, systematically extracted data about implementation issues from randomized controlled trials (RCTs) evaluating the use of PROMs. An RCT protocol design aims to ensure that the intervention is delivered as it is intended and also an outcome of successful implementation. Although not designed to measure implementation, RCTs have been purposefully designed to eliminate confounders and will report on these.

There is a lack of clear understanding of what influences successful PROM intervention implementation fidelity. This review may be useful to inform researchers on how to accurately measure implementation outcomes [13] and the implementation process [14].

### Objective

The primary objective of this systematic review is to identify and synthesize factors available from RCT data about the fidelity of PROM interventions in clinical practice. These factors will then be structured around the Promoting Action on Research Implementation in Health Services (iPARIHS) implementation science framework, such as those relating to the context, participants, the intervention itself, or the study team facilitating the research.

The primary objective will be to assess intervention fidelity and if this has any influence on study outcomes. The secondary objective will be to describe any factors impacting fidelity and the processes and outcomes associated with these.

### Research Question

This review will address the following research question:

What are the factors RCTs report that impact the implementation of PROMs into clinical practice?

## Methods

### Overview

This systematic review protocol has been registered in PROSPERO International Prospective Register of Systematic Reviews (CRD42017056138). It has been written according to the Preferred Reporting Items for Systematic Reviews and Meta-Analyses (PRISMA) protocols [15]. The checklist is included as Multimedia Appendix 1. The findings will be reported transparently [16,17] with a structured narrative synthesis [18]. If possible, a meta-analysis of implementation factors will be attempted.

### Criteria for Considering Studies for This Review

#### Types of Studies

This systematic review will include RCTs that used PROMs with feedback provided to clinicians to support their decision making in the clinical setting. The control will be standard clinical practice or usual care. RCTs have been chosen because their design ascertains the enrollment of a relatively homogenous group of patients that are balanced between the intervention and control groups and control for the impact of confounders. A review of RCTs offers an opportunity to study the fidelity of implementing the PROM intervention and factors associated with better implementation in an optimized setting.

The focus of this review is the use of PROMs as an intervention for patient care in clinical practice, and any study that does not use a PROM intervention will be excluded. RCTs that use proxy PROM measures (ie, those completed by a carer) will also be excluded, as this review focuses only on reports collected directly from the patient.

#### Types of Participants

Participants will be any patients attending health care facilities, including hospital outpatients, specialist clinics, and health centers as well as the staff caring for them in these facilities, that is, patients completing PROMs reports and the clinicians responding to PROMs information.

#### Types of Interventions

The intervention will be the completion of PROMs by patients, with the results fed back to clinical staff to use in their ongoing clinical care.

#### Types of Outcome Measures

The primary outcome is intervention implementation fidelity, that is, whether the intervention was delivered as it was intended or not. Intervention fidelity will be assessed using the criteria described in the systematic review by Proctor et al [13]: (1) adherence to the program protocol, (2) dose or amount of program delivered, and (3) quality of the program delivery.

### Search Methods for Identification of Studies

#### Electronic Searches

Searches of databases to be accessed for this step will include MEDLINE, EMBASE, and the Cumulative Index to Nursing and Allied Health Literature via the OvidSP website. All searches will be saved using an account established in each search database. Databases such as ClinicalTrials.gov will be searched for ongoing studies. Other sources of grey literature will be limited to clinical trial results reported in theses, dissertations, and conference papers. Field experts will be contacted.

#### Searching Other Resources

Field experts will be contacted for recommendations, and resources from the ISOQOL will be accessed. Reference lists of identified systematic reviews and studies will be searched to ensure a comprehensive list.

### Data Collection

The findings will be reported listing all outcomes in tables [17], summarized using a structured narrative synthesis [18], and a quantitative meta-analysis using forest plots (if possible) to present the data will be performed [16].

#### Selection of Studies

Studies will be screened initially by retrieving abstracts to identify whether they meet the eligibility criteria. Screening will be done independently by NR and MJ. If a study is identified as eligible for inclusion, the full-text version will be retrieved. After review of the full text, if a study is subsequently decided as not eligible, it will be excluded. A PRISMA flowchart will be used to present this information [15].

During the screening process, any discrepancies in the NR’s and MJ’s assessment of eligibility of the studies will be resolved by discussion. If this is not possible, the study will be referred to KA and DW for further input until a consensus is reached.

#### Data Extraction and Management

Endnote bibliographic software will be used to manage the literature by allocating studies using folders of *included*, *excluded*, and *for discussion*. Included studies will be entered for meta-analysis in RevMan 5.0. A database of the implementation data of included studies will be created during data extraction for narrative synthesis using Excel.

Data extraction will be done by NR and verified by MJ in 10% of studies. A data extraction table will be developed that, for each included study, states the research question, methodology, number of participants, primary outcome, secondary outcomes, tools used, and main findings to extract the demographics of the studies included.

**Figure 1:**
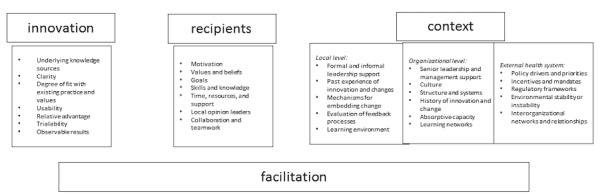
Components of the integrated Promoting Action on Research Implementation in Health Services framework.

Implementation data, including implementation strategies, process measurements, and outcome measurements will be extracted. The iPARIHS framework will be used to identify any implementation factors reported by study authors [19]. This information will be extracted using a content analysis approach. The key elements of iPARIHS are presented in Figure 1. The framework identifies what is present in the context for those exposed to the intervention and within the intervention itself. This framework also acknowledges the role of a facilitator, which has been identified as important [1]. RCTs often have research staff facilitating the study, so the impact of this can also be measured. This framework has been successfully used in a number of clinical studies, such as the prevention of Methicillin-resistant *Staphylococcus aureus* [20] and the Eat Walk Engage protocol [21]. If reported, these factors will be defined as impacting outcomes [13] or processes of implementation [14].

#### Assessment of Risk of Bias in Included Studies

##### Assessment of Methodological Quality and Risk of Bias

Other systematic reviews of efficacy have identified issues with study quality including attrition bias, performance bias, and a high risk of randomization bias for those health workers participating in the study [4,11]. Each study included will be assessed individually using the Cochrane risk of bias tool 2 (RoB 2.0).

This process will be carried out by the primary reviewers NR and MJ independently. If a consensus is needed, this will be addressed through discussion. If this is still not resolved, input will be sought from the other authors, KA and DW.

### Analysis

#### Measures of Treatment Effect, Unit of Analysis Issues, and Dealing With Missing Data

All eligible studies will be included in the narrative synthesis irrespective of the dose, delivery, duration, or intensity of the intervention, that is, independent of how often the PROM is administered, how it is administered, the duration of the PROM intervention and follow-up, and the number of PROMs administered.

If a meta-analysis is possible, it will be performed through a standard approach, that is, dichotomous data effect sizes will be calculated as odds ratios with 95% confidence interval, continuous data will be converted into standardized mean differences (SMDs), and time to event data will be calculated as hazard ratios. SMDs will be used to accommodate differences among PROM types reported by studies. If necessary, authors will be contacted if the report does not provide sufficient information. If the data cannot be obtained, the study will be excluded from the meta-analysis but still be included in the narrative synthesis. Similarly, studies with incomplete outcome data can still be included in the narrative synthesis if they report implementation factors.

#### Data Synthesis

A narrative data analysis approach [17,22] will be applied to the primary outcomes data collected based on the iPARIHS framework in Figure 1. This data will then be synthesized to describe intervention fidelity guided by the concepts presented in the study by Proctor et al [13]. The processes of implementation will be guided by the concepts described in the study by Powell et al [14]. Relationship within and between studies will be collated using mapping and tabulation of data [23]. The robustness of the synthesis will be assessed by comparing with previous systematic reviews, the scoping review, and study findings [18].

#### Meta-Analysis and Investigation of Heterogeneity

A meta-analysis will be performed to assess the impact of intervention fidelity on quality of life outcomes, provided there are sufficient data.

Stratified analysis of patient populations, for example, oncology, mental health, chronic diseases, will be attempted if there is heterogeneity in the data. Statistical heterogeneity will be assessed using I-square statistic to describe the variation across studies. There is likely to be heterogeneity in the evidence base because of enrolled patient populations or intervention design, and for that reason, a random effects model will be needed. The variability in effect estimates because of heterogeneity, rather than sampling error, will be classified as the proportion of observed effects [23]. This process is expected to allow quantitative consolidation of findings across studies.

#### Sensitivity Analysis

A sensitivity analysis will be done by removing studies at high risk of bias as assessed by RoB 2.0, and the impact on study outcomes will be reported [23].

## Results

This protocol has received funding. Searches of databases will commence at the end of May 2019. It is planned that this systematic review will be completed, and a paper will be finalized for publication in (December) 2019.

## Discussion

This systematic review will have some limitations that will be addressed where possible. Limiting to RCTs may result in mainly positive studies, but this will be accounted for in the final discussion of the systematic review. A search strategy confined to English language studies may result in a restricted assessment of health contexts and study findings. However, the search strategy is innovative, as the searches will extend beyond traditional databases and also include searches of clinical trial databases and consultation of experts in the field to capture any ongoing trials. The resulting systematic review can make a valuable contribution to the research knowledge.

PROMs have been shown to have the potential to improve both the processes of care and outcomes for patients. It is important to better understand how to translate these findings into clinical settings. This can ensure that the fidelity of future intervention can be improved, and outcomes can be achieved. An implementation science framework, such as iPARIHS, offers the opportunity in a systematic review to better identify the factors impacting on implementation. It is expected that the findings of this review can be used to describe the evidence available in RCTs investigating the use of PROMs in the clinical setting. This is important to rapidly progress PROMs in the clinical care of patients, as well as achieve better design and research studies in the future.

## Appendix

Multimedia Appendix 1 Peer-reviewer report from the National Health and Medical Research Council, Australian Government.

## Abbreviations

iPARIHS: Promoting Action on Research Implementation in Health Services
ISOQOL: International Society of Quality of Life
PRISMA: Preferred Reporting Items for Systematic Reviews and Meta-Analyses
PROM: patient-reported outcome measurement
RCT: randomized controlled trial
ROB: risk of bias
SMD: standardized mean difference
