# Supplementary material for: Factors in Randomized Controlled Trials Reported to Impact the Implementation of Patient-Reported Outcome Measures Into Routine Care: Protocol for a Systematic Review
Source: JMIR Res Protoc. 2019 Nov 26;8(11):e14579. doi: 10.2196/14579 (PMC6904897; doi:10.2196/14579)
Supplement: Multimedia Appendix 1 [file resprot_v8i11e14579_app1.pdf]

Australian Government

National Health and Medical Research Council

Application Assessment Summary

Postgraduate Scholarships

(for funding commencing in 2018)

Applicant's Name: Ms Natasha Roberts

Application ID: APP1151443

Administering Institution: Queensland University of Technology

Application Type: Public Health and Health Services Postgraduate Research Scholarship

The Peer Review Panel assessment of your application is summarised below:

**Assessment Criteria**	**Score**
Criterion 1 – Academic Record and Research Achievement – Relative to Opportunity (60%)	4.802
Criterion 2 – Research Environment and Supervisor (20%)	5.705
Criterion 3 – Research Project (20%)	5.028
**Overall Score**	**5.028**

**Your application fell in Quartile[1]**	**3**

The funding cut-off point fell at approximately 5.120 within Quartile 3. Quartiles were calculated against all applications.

[1] *Quartile 4 = Top Range of 0 to 25%*
Quartile 3 = Mid Range 25 to 50%
Quartile 2 = Lower Range 50 to 75%
Quartile 1 = Lowest Range 75 to 100%
